# Validation of Noninvasive MOEMS-Assisted Measurement System Based on CCD Sensor for Radial Pulse Analysis

**DOI:** 10.3390/s130405368

**Published:** 2013-04-22

**Authors:** Karolis Malinauskas, Paulius Palevicius, Minvydas Ragulskis, Vytautas Ostasevicius, Rolanas Dauksevicius

**Affiliations:** 1 Institute for Hi-Tech Development, Faculty of Mechanical Engineering and Mechatronics, Kaunas University of Technology, Studentu 65-209, Kaunas LT-51369, Lithuania; E-Mails: karolis.malinauskas@stud.ktu.lt (K.M.); vytautas.ostasevicius@ktu.lt (V.O.); 2 Research Group for Mathematical and Numerical Analysis of Dynamical Systems, Kaunas University of Technology, Studentu 50-222, Kaunas LT-51368, Lithuania; E-Mails: paulius.palevicius@stud.ktu.lt (P.P.); minvydas.ragulskis@ktu.lt (M.R.)

**Keywords:** radial pulse, laser triangulation, MOEMS, CCD sensor, projection moiré

## Abstract

Examination of wrist radial pulse is a noninvasive diagnostic method, which occupies a very important position in Traditional Chinese Medicine. It is based on manual palpation and therefore relies largely on the practitioner′s subjective technical skills and judgment. Consequently, it lacks reliability and consistency, which limits practical applications in clinical medicine. Thus, quantifiable characterization of the wrist pulse diagnosis method is a prerequisite for its further development and widespread use. This paper reports application of a noninvasive CCD sensor-based hybrid measurement system for radial pulse signal analysis. First, artery wall deformations caused by the blood flow are calibrated with a laser triangulation displacement sensor, following by the measurement of the deformations with projection moiré method. Different input pressures and fluids of various viscosities are used in the assembled artificial blood flow system in order to test the performance of laser triangulation technique with detection sensitivity enhancement through microfabricated retroreflective optical element placed on a synthetic vascular graft. Subsequently, the applicability of double-exposure whole-field projection moiré technique for registration of blood flow pulses is considered: a computational model and representative example are provided, followed by *in vitro* experiment performed on a vascular graft with artificial skin atop, which validates the suitability of the technique for characterization of skin surface deformations caused by the radial pulsation.

## 1. Introduction

**Figure 1.**
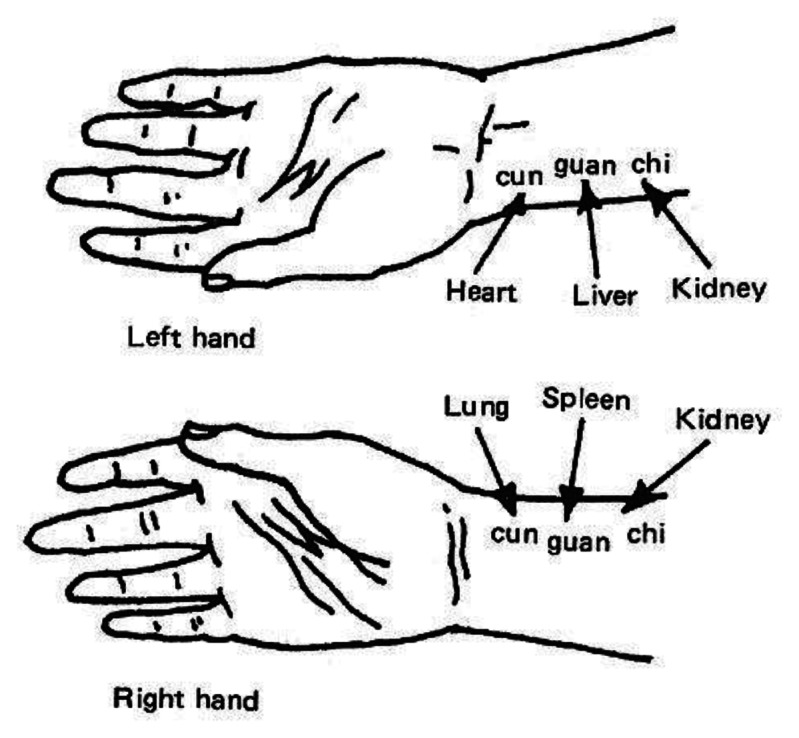
Different spots of radial artery on the wrist of the left and right hands represent different human organ in traditional Chinese pulse diagnosis.

Pulse diagnosis based on manual palpation has been practiced in Traditional Chinese Medicine for more than 2,000 years. In traditional Chinese pulse diagnosis (TCPD) theory, wrist radial pulse signals contain rich information that reflects the state of human health. The practitioner positions the fingertips at different wrist points along the radial artery (Figure 1) to feel patient′s pulse beating and then evaluates it according to various criteria (e.g., frequency, depth, quality, strength, rhythm) in order to determine the condition of different internal organs. Depending on the hand and sensing location on the wrist the practitioner can detect and predict abnormal symptoms and thereby identify the state of different organs [1].

The wrist pulse is considered to be the most fundamental signal of life, carrying essential information about person′s health. Pathologic changes in a body are reflected by fluctuation patterns of radial pulses. For example, clinical studies indicate that patients with hypertension, cardiovascular disease and diabetes exhibit premature loss of arterial elasticity. Pulse shape, amplitude and rhythm also undergo changes as a result of variations in hemodynamic characteristics of blood flow [1,2].

It is universally acknowledged that development of effective preventive medical systems is crucial for contemporary healthcare therefore Chinese pulse diagnosis occupies an important position in this respect [3]. However, TCPD results are heavily dependent on the practitioner′s subjective skills and experience, thereby making questionable the accuracy and reliability of the method. Moreover, classifications of pulse patterns proposed by different Chinese physicians are ambiguous. The problem is that TCPD have not progressed beyond the stage of manual palpations therefore modern sensor devices and measurement techniques need to be adapted for quantified description of the Chinese pulse diagnostic method in order to make it objective and enable its further advancement.

The aim of this research work is to investigate the applicability of optical noninvasive measurement techniques for registration of radial blood flow pulses. The paper is organized as follows: radial pulse waveforms are discussed in Section 2, followed by the calibration of the pulses in the artificial artery by means of a laser triangulation sensor in Section 3. The projection moiré method for investigation of surface deformations is presented in Section 4 and the applicability of the method for the registration of blood flow pulses is considered in Section 5. Concluding remarks are provided in the final section.

## 2. Radial Pulse Characteristics

**Figure 2.**
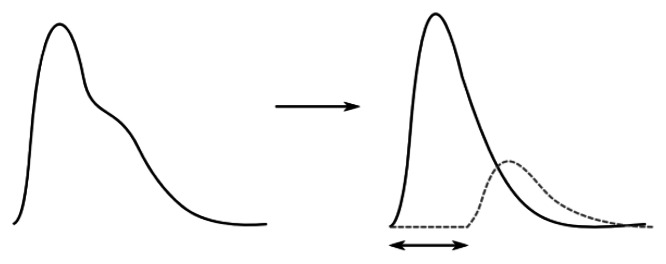
Forward pulse wave is higher in amplitude, while backward wave is lower in amplitude and shifted in phase.

A beating heart generates pressure and flow waves that propagate throughout the arterial system. The shapes of pulse waveforms are modified by their continuous interaction with the non-uniform arterial tree. The pressure waves expand the arterial walls when traveling, thereby inducing wrist pulses, which can be distinguished in terms of one forward traveling wave component (collective waves that spread out from heart to periphery and provide information about the heart itself) and one backward traveling wave component (collective waves carrying information of the reflection sites, *i.e.*, kidney, stomach, spleen, liver, lungs, *etc.*). In addition, the reflected pressure waves tend to augment the load on the heart and are decisive in determining wrist pulse waveform patterns [4,5]. Thus, wrist pulse waveforms can be expressed in terms of its forward and backward traveling components with a phase shift in time (Figure 2).

**Figure 3.**
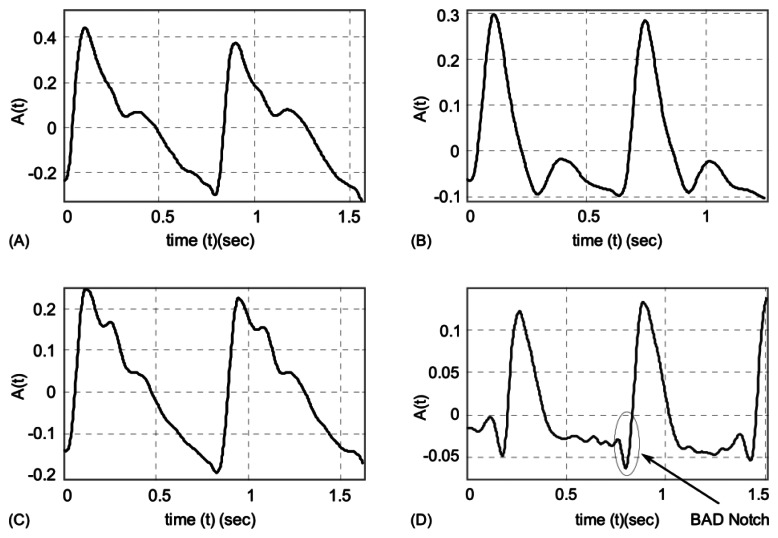
(**A**–**C**) pulse patterns of young healthy persons: (A) taut, (B) slippery, (C) moderate. Plot (**D**) illustrates abnormal pulse pattern containing BAD Notch.

A normal wrist pulse waveform is characterized by a smooth and sharp upstroke, a short peak, followed by a quick downstroke and decay. The reflected wave resembles the initial wave in terms of shape but has lower amplitude. Figure 3(A–C) illustrate pulse patterns that are typical of young healthy people. In accordance with the Traditional Chinese Medicine terminology these graphs clearly reveal the presence of dicrotic notch and dicrotic wave and the pulses are identified as *taut*, *slippery* or *moderate*. The abnormal pulse pattern in Figure 3(D) is characterized by formation of a unique V-shaped notch referred to as BAD Notch [6].

## 3. Optical Calibration of Radial Blood Flow Pulses

In general, a computerized pulse signal diagnosis involves three major stages: data collection, feature extraction and pattern classification. In this research work we consider only data collection aspect with the aim to obtain measurement data required for calibration of artery displacements.

**Figure 4.**
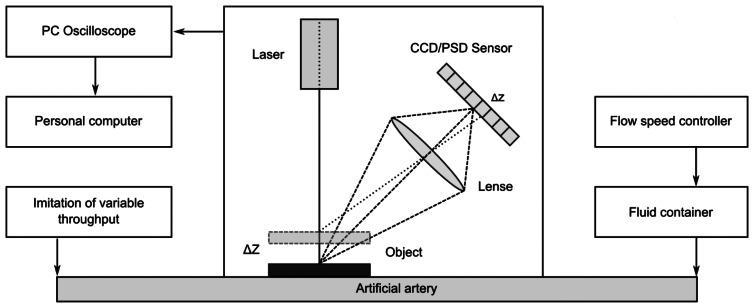
Scheme for measurement of artificial radial pulses by means of laser triangulation technique.

**Figure 5.**
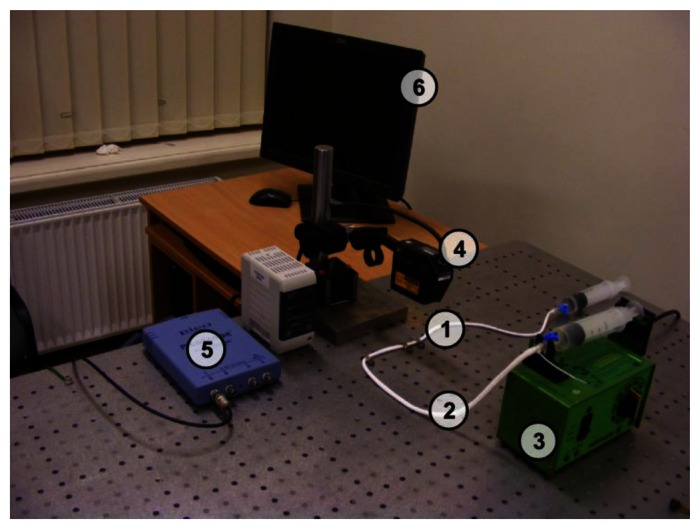
Experimental setup for registration of artificial radial blood flow pulsation: (1) measurement location (test point); (2) vascular graft; (3) flow speed controller; (4) laser triangulation sensor; (5) digital oscilloscope; (6) PC for data management.

Figures 4–5 provide a scheme and photo of the experimental setup that was used for *in vitro* measurements of artificial blood flow pulsation by laser triangulation technique. Artificial artery [commercially-available 2.4 mm Ø vascular graft intended for surgical use) was employed in the setup for registration of pulse waveforms. It is made of expanded polytetrafluoroethylene (ePTFE, Young′s modulus—600 MPa) consisting of a carbon and fluorine based synthetic polymer that is biologically inert and non-biodegradable in the body. A simplified closed-loop fluid flow system with input valve and output pressurized chamber (generating variable fluid throughput) was set up for imitating blood flow in radial artery. A flow speed controller was installed in order to regulate the flow rate in the range from 50 mL/h to 600 mL/h. Pulse waves are generated by variation of the flow rate, which leads to different output pressure values in the system. The displacements of a test point on the vascular graft were measured by means of a KEYENCE LK-G82 high-accuracy laser triangulation CCD displacement sensor with a sampling speed of 50 kHz, accuracy of ±0.02% and repeatability of 0.01 μm. A retroreflective optical element [a membrane microfabricated by using technology of micro-opto-electro-mechanical systems (MOEMS)] was attached onto the vascular graft for maximizing the amount of the back-scattered laser light collected by the CCD sensor (the membrane is intended for the use inside a portable wrist-worn sensor device as a transducer element). Measurement data was transmitted to the computer for analysis via analog-to-digital converter (PICO 3424 oscilloscope). PicoScope software was used for examination of the registered pulse waveforms.

**Figure 6.**
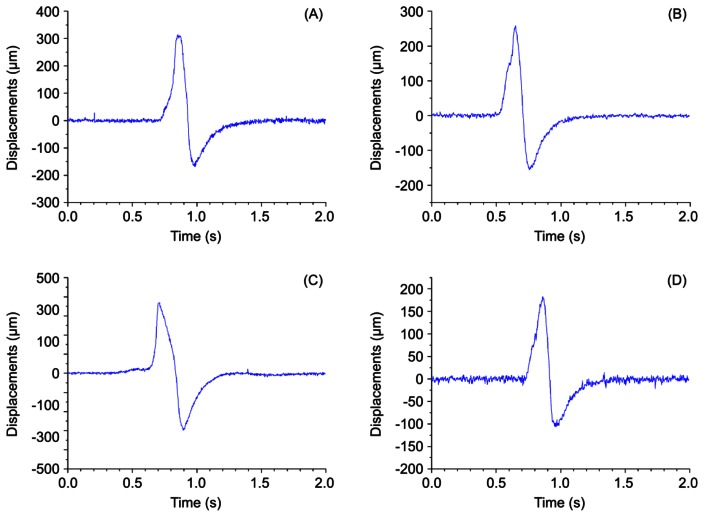
Plots of registered data for different values of pressure applied to artificial blood flow system: (**A**), (**C**) 120 mmHg; (**B**), (**D**) 140 mmHg ((A), (B) artificial blood; (C), (D) water).

In the presented laser triangulation optical setup, the movement of the MOEMS membrane caused by a pulsating artery leads to a modification of the laser beam projection on the CCD sensor, thereby enabling registration of a pulse shape. During experimental study the artificial blood flow system was subjected to two different pressures: 120 mmHg (∼16 kPa) and 140 mmHg (∼18.7 kPa). The first value corresponds to a normal systolic pressure of a healthy person and the second value is characteristic of a person with a possible hypertension condition. Displacements of a test point were registered for two cases (Figure 6): (a) when lower viscosity fluid (water) is used (8.9 × 10^−4^ Pa·s), (b) when higher viscosity artificial blood is used (3.2 × 10^−3^ Pamiddot;s). Measurement results in Figure 6 indicate that higher displacements of the test point are recorded when a more viscous fluid is introduced into the vascular graft. The plots also reveal that qualitatively the artificial pulse waveforms roughly resemble the shape of actual pulses (Figure 3). It is understandable that exact reproduction of variation patterns of actual pulses is hardly possible during *in vitro* experiments. Quantitatively, the absolute value of amplitude of the artificial pulses is comparable to that of actual pulses (amplitude of radial pulses varies in a wide sub-millimeter range).

Obtained experimental results confirm that blood pulse fluctuations may be accurately traced by applying laser triangulation sensor that is used in conjunction with the retroreflective MOEMS membrane placed onto the artery for improving displacement detection sensitivity.

## 4. The Projection Moiré Method for Investigation of Surface Deformations

The projection moiré technique allows obtaining the relief of an object [7–9]. The classical application of the projection moiré method for measurement of out-of-plane displacements by the difference in relief between two prompting states will be used in this section. The displacement field can be extracted from the fringe pattern produced by double exposure of images in both states.

### 4.1. Mathematical Representation of the Projected Image

It should be noted that a paraxial model is used in the following steps (this condition can be approximated by using a slide projector that is located far from the specimen and by placing the imaging system far away from the specimen). An important factor, which must be considered in practical applications, is the problem of depth of focus. A camera can focus one plane only; all the other points in the surface under analysis experience a change of coordinates that introduces an error. As the paraxial model is adopted here, it is not necessary to deal with the problem of depth of focus and perspective effects caused by a point light source.

**Figure 7.**
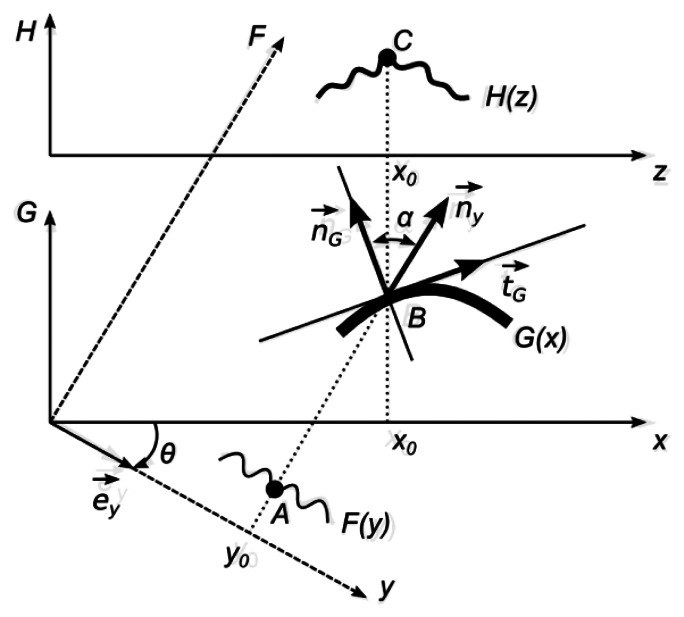
One-dimensional geometrical representation of optical projection on a diffuse surface. *F(y)* projected image, *G(x)* diffuse deformed surface, *H(z)* observed image.

It is assumed that the direction of observation is perpendicular to the *x*-axis and the angle between the direction of illumination and the direction of observation is θ. One-dimensional geometrical representation of the optical projection on a diffuse surface is provided in Figure 7.

The projected image *F(y)* is defined in a frame *y0F*, which is rotated with respect to frame *x0G* by an angle *θ*. The function *F(y)* determines a greyscale level of a white light ray travelling through a point *y_0_* along the *F*-axis. Therefore, it is assumed that 0 ⪯ *F(y)* ⪯ 1, where 0 represents the black color, 1—white color, and all intermediate values stand for appropriate greyscale levels. The functional illustration of *F(y)* is given in Figure 7 since it makes the construction of geometrical relationships easier:

(1) $${\vec{e}}_{y}=\left(\cos\theta ;\sin\theta \right)$$

(2) $${\vec{n}}_{y}=\left(-\sin\theta ;\cos\theta \right)$$

(3) $${\vec{t}}_{G}=\left(\frac{1}{\sqrt{1+{\left(G_{x}^{\prime }\left(x\right)\right)}^{2}}};\frac{G_{x}^{\prime }\left(x\right)}{\sqrt{1+{\left(G_{x}^{\prime }\left(x\right)\right)}^{2}}}\right)$$

(4) $${\vec{n}}_{G}=\left(-\frac{G_{x}^{\prime }\left(x\right)}{\sqrt{1+{\left(G_{x}^{\prime }\left(x\right)\right)}^{2}}};\frac{1}{\sqrt{1+{\left(G_{x}^{\prime }\left(x\right)\right)}^{2}}}\right)$$

As mentioned previously, the surface *G(x)* is a diffuse surface. Therefore, the observed greyscale level at a point *z* is:

(5) H(z)=H(x):=F(y)cosα

(6) $$\cos\alpha =\left({\vec{n}}_{y}\cdot {\vec{n}}_{G}\right)=\frac{\cos\theta +G_{x}^{\prime }\left(x\right)\sin\theta }{\sqrt{1+{\left(G_{x}^{\prime }\left(x\right)\right)}^{2}}}$$

The following equality holds for all positive *x* and *y*:

(7) y=xcosθ−G(x)sinθ

Thus, finally:

(8) $$H\left(x\right)=F\left(x\cos\theta -G\left(x\right)\sin\theta \right)\frac{\cos\theta +G_{x}^{\prime }\left(x\right)\sin\theta }{\sqrt{1+{\left(G_{x}^{\prime }\left(x\right)\right)}^{2}}}$$

This equation gives an exact description of the image formation process.

### 4.2. Double-Exposure Projection Moiré

The double-exposure projection moiré technique comprises two steps. Initially, the grating is projected obliquely to the viewing direction on a surface *G(x)* and the observed grating is photographed. Then, the specimen is deformed (the grating projection and imaging systems remain unchanged) and the observed grating is photographed again. Superposition of these two images produces moiré fringes, which can be used to identify the magnitude of specimen deformation.

The surface of the deformed specimen can be described as *G(x)*+*g(x)*, where *g(x)* is the absolute deformation of the specimen in the direction of observation after the load was applied. We assume that the projected image is a harmonic moiré grating:

(9) $$F\left(y\right)=\frac{1}{2}+\frac{1}{2}\cos\left(\frac{2\pi }{\lambda }y\right)$$

where *λ* is the pitch of the grating. Moreover, we will assume that the function *g(x)* is a slowly varying function. In other words, we require that:

(10) $$\frac{\cos\theta +\left(G_{x}^{\prime }\left(x\right)+G_{x}^{\prime }\left(x\right)\right)\sin\theta }{\sqrt{1+{\left(G_{x}^{\prime }\left(x\right)+G_{x}^{\prime }\left(x\right)\right)}^{2}}}\approx \frac{\cos\theta +G_{x}^{\prime }\left(x\right)\sin\theta }{\sqrt{1+{\left(G_{x}^{\prime }\left(x\right)\right)}^{2}}}$$

Then, subtractive superposition of the observed grating before and after the load produces:

(11) $$\begin{array}{c} \frac{1}{2}+\frac{H_{1}\left(x\right)-H_{2}\left(x\right)}{2}\times \frac{\sqrt{1+{\left(G_{x}^{\prime }\left(x\right)\right)}^{2}}}{\cos\theta +G_{x}^{\prime }\left(x\right)\sin\theta }\approx \\ \frac{1}{2}+\frac{1}{4}\left(\cos\frac{2\pi }{\lambda }\left(x\cos\theta -G\left(x\right)\sin\theta \right)-\cos\frac{2\pi }{\lambda }\left(x\cos\theta -\left(G\left(x\right)+g\left(x\right)\sin\theta \right)\right)\right)=\\ \frac{1}{2}-\frac{1}{2}\left(\sin\frac{2\pi }{\lambda }\left(x\cos\theta -G\left(x\right)\sin\theta -\frac{g\left(x\right)\sin\theta }{2}\right)\right)\times \sin\left(\frac{2\pi }{\lambda }\frac{g\left(x\right)\sin\theta }{2}\right) \end{array}$$

where *H _1_ (x)* and *H_2_ (x)* are observed gratings before and after the load. The previous equation represents the effect of beats. The envelope function is:

(12) $$\frac{1}{2}\pm \frac{1}{2}\sin\left(\frac{2\pi }{\lambda }\frac{g\left(x\right)\sin\theta }{2}\right)$$

Moiré fringes will form at:

(13) $$\frac{\pi g\left(x\right)\sin\theta }{\lambda }=\pi N;N=0,\pm 1,\pm 2,...$$

Finally, the displacement *g(x)* in terms of fringe order *N* reads as:

(14) $$g\left(x\right)=\frac{\left(0.5+N\right)\lambda }{\sin\theta }$$

### 4.3. Two-Dimensional Example

**Figure 8.**
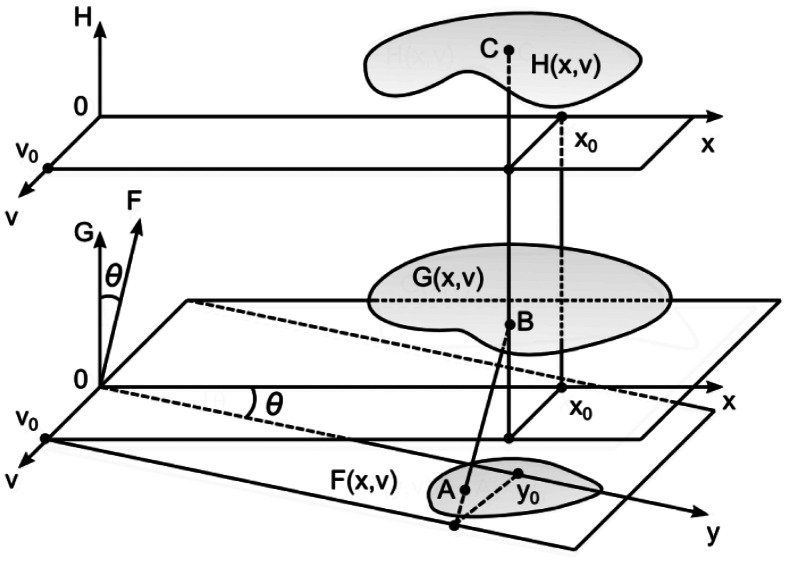
Schematic representation of the projection process.

In general, the image plane, the surface plane and the observation plane can be located independently in the three-dimensional space. As it is assumed that the angle of observation is zero, the *H*-plane and the *G*-plane are parallel (Figure 8). The angle of illumination is *θ*. Thus, it is assumed (without loss of generality) that the angle between the *F*-plane and the *G*-plane is also *θ*. As previously, the function *F(y,v)* determines greyscale levels of the projected image, *G_K_ (x,v)*—the shape of the surface, *H_K_ (x,v)*—greyscale levels of the observed image. Then:

(15) $$F\left(x,v\right)=\frac{1}{2}+\frac{1}{2}\cos\left(\frac{2\pi \left(x\cos\alpha -v\sin\alpha \right)}{\lambda }y\right)$$

(16) $$H_{K}\left(x,v\right)=F\left(x\cos\theta -G\left(x,v\right)\sin\theta ,v\right)\frac{\cos\theta +\frac{\partial G_{K}\left(x,v\right)}{\partial x}\sin\theta }{\sqrt{1+{\left(\frac{\partial G_{K}\left(x,v\right)}{\partial x}\right)}^{2}}}$$

## 5. The Applicability of Whole Field Projection Moiré for the Registration of Radial Pulses

### 5.1. Computational Example

**Figure 9.**
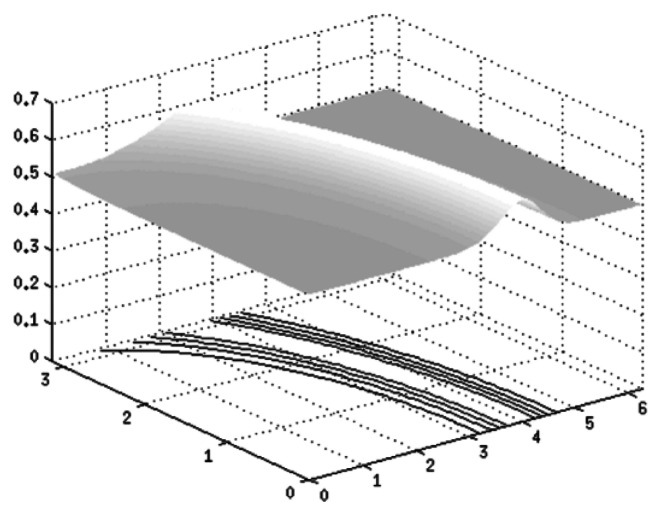
Illustration of surface described by function *G(x)*.

A surface of skin with an artery going underneath is given in Figure 9. This surface is described by the function *G_K_ (x,v)*:

(17) $$G_{K}\left(x,v\right)=0.1\exp\left(-0.05{\left(x^{2}+v^{2}-2^{2}\right)}^{2}\right)$$

**Figure 10.**
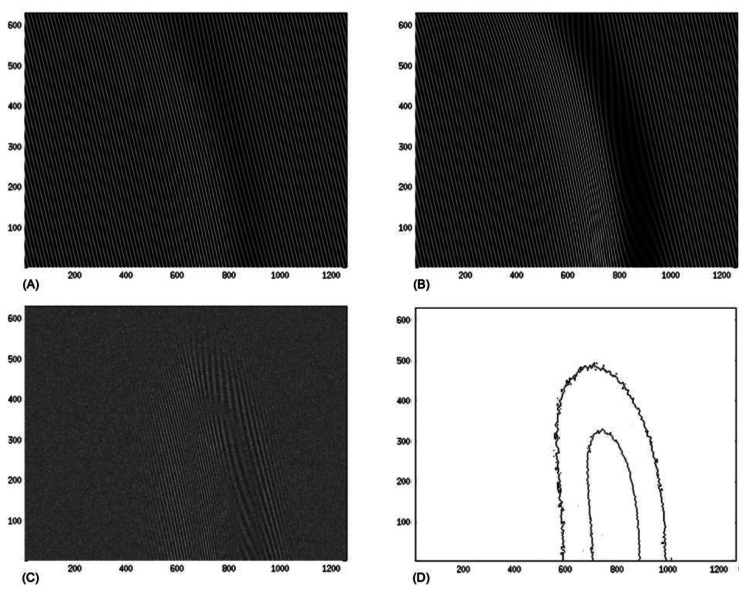
(**A**), (**B**) projected grating on a surface *G(x)* and deformed surface, respectively; (**C**) subtractive superposition of (**A**) and (**B**); **(D)** contour lines of the deformation.

The angle of illumination is *θ* = 45° and grating pitch is λ = 0.05. An impulse of pressure is applied to a given surface, which results in a deformation of initial relief. Figure 10(A) shows a projected grating on a given surface *G(x)* and (B) illustrates a projected grating on a deformed surface *G(x)*. Subtractive superposition of those surfaces is provided in image (C). Image (D) presents contour lines of the deformation.

### 5.2. Experimental Setup

**Figure 11.**
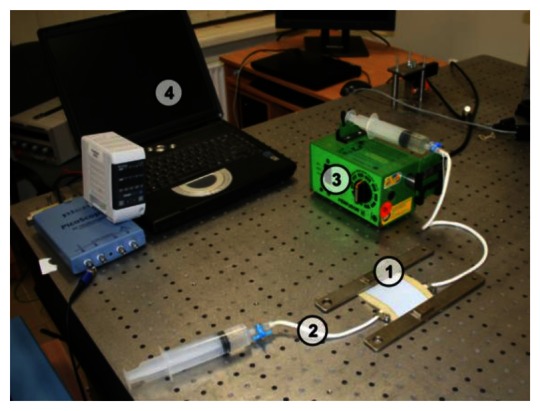
Experimental setup for registration of artificial radial pulses: (1) measurement location (test point); (2) vascular graft; (3) flow speed controller; (4) data registration and presentation.

Experimental setup for registration of artificial radial pulses is provided in Figure 11. The artificial artery is placed under a deformable layer of polyurethane of 2 mm thickness and is covered by a thin deformable rubber membrane. Such a setup mimics human tissue and skin over an artery located near the surface of the skin. A radial pulse of the artificial artery generated by the non-uniform blood flow induces deformation of the artificial skin (and hypodermic layer). Such skin deformation can be measured by the double-exposure whole-field projection moiré.

**Figure 12.**
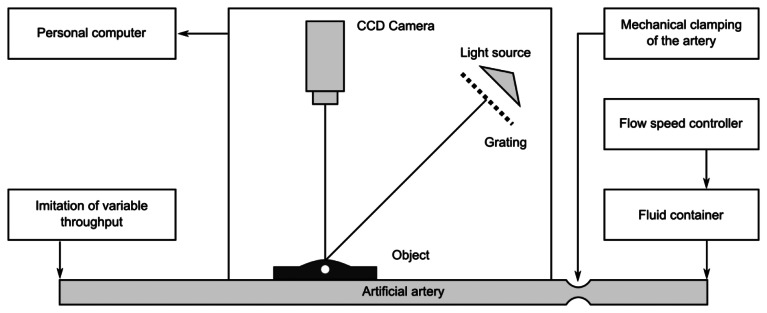
Scheme for measurement of artificial radial blood flow pulsation by means of double-exposure whole-field projection moiré.

**Figure 13.**
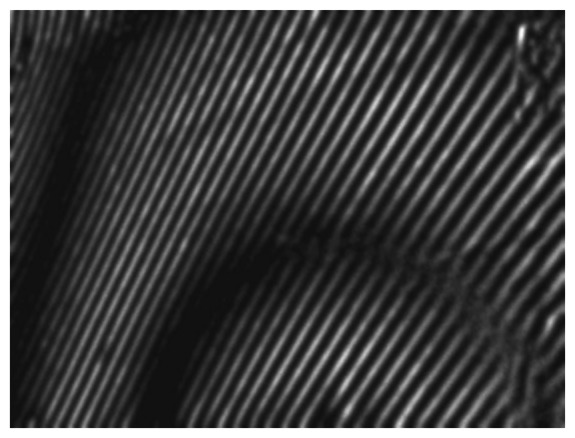
Result of the double-exposure whole-field projection moiré.

The scheme of the whole-field projection moiré optical setup is illustrated in Figure 12. Initially, the surface of the artificial skin is illuminated at 45°. Camera continuously captures images of the projected grating. Superposition of these images provides a result shown in Figure 13.

## 6. Conclusions

The paper proposed a noninvasive optical measurement system for characterization of radial pulse waveforms, which is based on a complementary application of laser triangulation and projection moiré techniques implemented by means of a CCD displacement sensor. A retroreflective MOEMS membrane placed on the pulsating artificial artery was successfully used to increase the sensitivity of displacement measurements performed with a laser triangulation CCD sensor. *In vitro* experiments conducted with a synthetic vascular graft have confirmed that laser triangulation sensor assisted by the MOEMS membrane enables high-sensitivity detection of artery displacements caused by variations in blood pressure. Further development of radial pulse monitoring methodology would involve filtering out the noise that is present in the registered pulse signals, while subsequent application of appropriate pattern matching methods would allow establishing various radial pulse parameters on the basis of TCPD theory.

A double-exposure projection moiré technique was proposed for the evaluation of skin surface deformations that are induced by pulsation of radial artery. A computational model was derived and used to calculate a two-dimensional deformation profile of the surface when it is subjected to pressure impulse. The applicability of the double-exposure whole-field projection moiré technique for pulse registration was experimentally verified by measuring deformation of the artificial skin with vascular graft underneath when pressure is varied in the artificial blood flow system.

The presented hybrid single-point/whole-field radial pulse characterization methodology that is jointly used with the computational model of projection moiré, constitutes an accurate noninvasive measurement tool that is able to collect reliable data on pulse fluctuations, which would be highly valuable for subsequent derivation of important hemodynamic parameters such as heart rate, arterial pressure and blood viscosity.
